# Distinct B cell profiles characterise healthy weight and obesity pre- and post-bariatric surgery

**DOI:** 10.1038/s41366-023-01344-y

**Published:** 2023-07-18

**Authors:** B. Šlisere, M. Arisova, O. Aizbalte, M. M. Salmiņa, M. Zolovs, M. Levenšteins, M. Mukāns, I. Troickis, L. Meija, A. Lejnieks, G. Bīlande, E. C. Rosser, K. Oļeiņika

**Affiliations:** 1 Department of Doctoral Studies, Riga Stradins University, Riga, Latvia; 2 Joint Laboratory, Pauls Stradins Clinical University Hospital, Riga, Latvia; 3 Department of Human Physiology and Biochemistry, Riga Stradins University, Riga, Latvia; 4 Department of Sports and Nutrition, Riga Stradins University, Riga, Latvia; 5 Daugavpils Regional Hospital, Daugavpils, Latvia; 6 Institute of Public Health, Riga Stradins University, Riga, Latvia; 7 Statistics Unit, Riga Stradins University, Riga, Latvia; 8 Institute of Life Sciences and Technology, Daugavpils University, Daugavpils, Latvia; 9 Aiwa clinic, Riga, Latvia; 10 Department of Internal Diseases, Riga Stradins University, Riga, Latvia; 11 Riga East University Hospital, Riga, Latvia; 12 Faculty of Medicine, University of Latvia, Riga, Latvia; 13 Centre for Adolescent Rheumatology Versus Arthritis at UCL, UCLH and GOSH and Centre for Rheumatology Research, Division of Medicine, UCL, London, UK; 14 Program in Cellular and Molecular Medicine, Boston Children’s Hospital, Harvard Medical School, Boston, MA USA

**Keywords:** Immunology, Homeostasis, Inflammatory diseases, Obesity

## Abstract

**Background/Objectives:**

Obesity-associated metabolic dysfunction and inflammation can be ameliorated by bariatric surgery. While obesity is also linked to impaired B cell activation, differentiation, and persistence in response to infection and vaccination little is known about post-operative immune B cell compartment and to what extent dysregulation in B cell pathways can be reversed. To bridge this gap in knowledge, we carried out in-depth evaluation of B cell composition in individuals with obesity prior to and following bariatric surgery compared to lean controls.

**Subjects/Methods:**

We recruited individuals with obesity (BMI at least 35 kg/m^2^) before bariatric surgery (*n* = 21) and followed them up 6 months post-operatively (*n* = 17). As controls we recruited age- and sex-matched lean (BMI < 25) individuals (*n* = 18). We carried out comprehensive immunophenotyping of peripheral blood B cells as well as interrogated their association with inflammatory and metabolic parameters.

**Results:**

In obesity the balance of antigen-inexperienced and memory B cells in the peripheral blood is altered, with an expansion of naïve and a reduction in total memory B cells. 6 months following bariatric surgery this balance is restored. However, post-operative patients are uniquely characterised by an increase in B cell subsets associated with chronic inflammation – CD11c^+^CXCR5^-^IgD^-^CD27^-^ double negative 2 (DN2) B cells and CD27^+^CD38^++^ plasmablasts. Correlations between B cells subsets, inflammatory and metabolic parameters were distinct in lean people and individuals with obesity pre- and post-bariatric surgery.

**Conclusions:**

Bariatric surgery patients display a unique B cell profile 6 months post-operatively; this bears minimal resemblance to that of pre-operative patients and only partially overlaps with that of lean controls. Post-operative differences in the B cell compartment compared to lean controls are detected despite global amelioration of inflammation and restoration of metabolic health. Collectively, this indicates that bariatric surgery creates a specific immunometabolic state with potential implications for health outcomes.

## Introduction

Obesity, defined as body-mass index (BMI) ≥ 30 kg/m^2^, is associated with chronic low-grade inflammation and leukocyte defects [1]. Leukocyte defects in obesity are postulated to not only exacerbate metabolic dysfunction, but also to impair immunity, including anti-pathogen and vaccination responses [2, 3]. Indeed, obesity is a risk factor for COVID-19 infection, morbidity, and mortality [4, 5]. Bariatric surgery, indicated as an intervention for obesity [6], is associated with significant and sustained weight loss, as well as improved metabolic health and reduction in circulating inflammatory factors long-term [7–11]. More recent studies have started to address the impact of bariatric surgery on immune compartments [12–15].

B cells play a central role in the protection against pathogens and the resolution of inflammation. B cell subsets are defined based on surface marker expression, which reports maturation stage, previous antigen experience as well as their differentiation and functional potential; for example, the propensity to rapidly generate class-switched antibody-secreting cells [16]. In healthy individuals, the peripheral blood B cell compartment is composed of CD24^hi^CD38^hi^ transitional, CD24^int^CD38^int^ mature B cells, IgD^+^CD27^+^ unswitched and IgD^-^CD27^+^ switched memory B cells, as well as a small population of IgD^-^CD27^-^ double-negative (DN) B cells [17, 18]. DN B cells can be further distinguished as CXCR5^+^CD11c^-^ DN1 and CD11c^+^CXCR5^-^ DN2 subsets. DN1 cells, expressing the B cell follicle homing receptor CXCR5, compose the majority of DN cells in healthy individuals and are considered recent emigrants of germinal centre reactions [19]. Conversely, DN2 B cells are thought to derive from extrafollicularly activated B cells, which support rapid inflammatory responses [19], and are substantially expanded in patients with active systemic lupus erythematosus (SLE), correlating with disease activity and poorer outcomes [17]. More recently, DN2 B cells have also been implicated in COVID-19 morbidity [20] as well as its post-acute sequalae [21]. Emergence of CD27^+^CD38^++^ plasmablasts in peripheral blood can further be used as a measure of on-going B cell activation.

Only a limited number of studies have investigated select aspects of B cell activation in individuals with obesity and there is even greater scarcity of investigations into the impact of bariatric surgery and weight loss on the B cell compartment. Obesity-associated alterations include an increase in mature naïve B cells [12, 22] as well as a reduction in total and IL-10 producing transitional B cells [23]. Notably, IL-10 production is a key phenotypic feature of B cells with immunoregulatory function [24]. Reports defining how DN B cell frequency is impacted by obesity remain inconsistent.; Frasca et al. reported an obesity-associated expansion in DN B cells, while Wijngaarden et al. instead found no differences in DN B cell frequency between lean people and individuals with obesity before bariatric surgery and 3 months post-bariatric surgery [12, 22].

Here we set out to comprehensively characterise obesity-associated peripheral blood B cell profiles compared to lean controls and to uncover the impact of bariatric surgery on the B cell compartment in paired samples from the same individual pre- and post-bariatric surgery. While we found that obesity-associated reduction in CD24^int^CD38^int^ memory B cells and expansion of IgD^+^CD27^-^ naïve B cells was reversed post-bariatric surgery, CD11c^+^CXCR5^-^IgD^-^CD27^-^ DN2 B cells and CD27^+^CD38^++^ plasmablasts associated with chronic inflammatory activation were expanded in post-bariatric patients compared to lean controls. Our data further demonstrate that correlations observed between inflammatory and metabolic parameters and B cell subsets in lean individuals are lost during obesity and are unique 6 months post-bariatric surgery. Collectively, these data establish that lean people and individuals with obesity pre- and post- bariatric surgery have distinct immunometabolic states that could have potential implications on their immediate and long-term health.

## Methods

### Study participants

People with obesity immediately prior to undergoing bariatric surgery at Aiwa Clinic were enrolled in the study between December 2020 and December 2021; 20 of recruited patients underwent Roux-en-Y gastric bypass (RYGB), 1 had sleeve gastrectomy (SG). To be eligible for RYGB or SG, patients had to meet the guidelines of the European Association for Endoscopic Surgery (EAES) surgery: update 2020 endorsed by IFSO-EC, EASO and ESPCOP; that is, BMI≥ 40 kg/m^2^ or BMI≥ 35 kg/m^2^ and at least one serious comorbidity, including, hypertension, type 2 diabetes, obstructive sleep apnoea, gastroesophageal reflux disease, polycystic ovary syndrome [25]. Age- and sex-matched lean (BMI < 25 kg/m^2^) control subjects were recruited. Adults that were < 60 years old were selected to minimise confounding effects from low-grade inflammation and immune senescence associated with aging [26]. Individuals with a history of malabsorptive or restrictive intestinal surgery, current pregnancy or lactating, severe organ dysfunction, cardiovascular disease, renal or hepatic diseases, acute or chronic inflammatory diseases, autoimmune diseases, infectious diseases, immunodeficiency, malignancies, substance and alcohol abuse, smokers as well as individuals taking immunomodulatory medication were excluded from the study. We further excluded individuals with uncontrolled hypertension and serum triglyceride concentration ≥ 4.5 mmol/l. All participants provided written informed consent. This study was approved by the Research Ethics Committee of Riga Stradins University (Riga, Latvia), and performed under the guidance of Declaration of Helsinki.

### PBMC isolation and serum collection

Peripheral blood was obtained from individuals with obesity prior to bariatric surgery, as well as at 6 months post-surgery and from lean controls at a single time-point. Overnight fasting was strictly enforced to avoid postprandial leukocyte activation. Serum samples from tubes with coagulation activator were isolated by centrifugation and serum used immediately or stored at −80 °C long-term. Tubes with ethylenediaminetetraacetic acid were used for full blood count and HbA1c determination. Tubes with sodium fluoride/potassium oxalate were used for plasma glucose assessment. PBMCs were isolated from heparinized blood by density gradient centrifugation using Histopaque-1077 (Sigma-Aldrich, MO, USA). After washing with complete RPMI-1640 medium (10% fetal bovine serum and 1% penicillin-streptomycin in RPMI-1640), PBMCs were re-suspended in freezing media (90% fetal bovine serum and 10% dimethyl sulfoxide and frozen in the Nalgene Mr. Frosty (Sigma-Aldrich, MO, USA) freezing container in −80 °C, and subsequently cryopreserved. All laboratory analyses were done in the Joint Laboratory at Pauls Stradins Clinical University Hospital (Riga, Latvia).

### Immunophenotyping of peripheral blood B cells by flow cytometry

CD27 and IgD allowed to assess naïve (IgD^+^CD27^-^), class-switched (IgD^-^CD27^+^) and unswitched (IgD^+^CD27^+^) memory and double negative (IgD^-^CD27^-^) B cells; CXCR5^+^CD11c^-^IgD^-^CD27^-^ DN1 and CD11c^+^CXCR5^-^IgD^-^CD27^-^ DN2 subset frequencies were also interrogated. Using CD24/CD38 expression, we assayed the frequencies of transitional (CD24^hi^CD38^hi^), mature (CD24^int^CD38^int^), memory (CD24^hi^CD38^lo^) and activated B cells (CD24^lo^CD38^lo^) as well as pre-plasmablasts (CD24^lo^CD38^hi^). Plasmablast (CD27^+^CD38^++^) populations were assessed in CD19^+^ B cells, gating them out prior to B cell analysis. Briefly, for PBMCs viability LIVE/DEAD Fixable Near-IR Dead Cell Stain Kit was used (Invitrogen, MA, USA). Nonspecific staining was prevented with Fc-receptor blocking reagent (Miltenyi Biotec, Bergisch Gladbach, Germany). The cells were incubated with antibodies (details see Suppl. Table) for 40 min at 4 °C, afterwards unbound antibodies were removed by two washes with flow cytometry staining buffer (2% fetal bovine serum and 2 mM ethylenediaminetetraacetic acid in phosphate-buffered saline) and fixed with phosphate-buffered saline containing 2% formaldehyde. Immunophenotypic studies were performed using the Navios EX flow cytometer (Beckman Coulter, Inc., Brea, CA, USA) and analysed with FlowJo software (BD Life Sciences). At least 1 million total PBMCs were stained and analysed by flow cytometry per sample.

### Assessment of metabolic profile and inflammatory markers

Plasma glucose and HbA1c were measured using a Cobas Integra 400 plus analyser (Roche Diagnostics, Manheim, Germany). Serum insulin and lipid profile were measured using a Siemens Atellica system (Siemens Healthineers, Erlangen, Germany). HOMA-IR (homeostatic model assessment for insulin resistance) index was calculated using the following formula: Glucose (mmol/L) × Insulin (mU/L) / 22.5. ELISA was used to determine serum concentrations of leptin (cat no KAC2281, Invitrogen, UK), adiponectin (cat no KHP0041, Invitrogen, Vienna, Austria), high sensitivity C-reactive protein (CRP) (cat no EU59151, IBL-International GmbH, Hamburg, Germany) according to the manufacturer’s instructions.

### Complete blood count

Blood counts were done with a UniCel DxH 800 hematology analyzer (Beckman Coulter Inc., Brea, CA, USA) and erythrocyte sedimentation rate (ESR) by capillary photometry method using a Roller 20-LC (Alifax, Padua, Italy).

### Statistical analysis

All the statistical analyses were conducted using GraphPad Prism 9 (La Jolla, CA, USA). Data distribution was assessed by the Shapiro-Wilk test and normal Q-Q plots. For normally distributed and homogenous data, independent samples t-test was used to compare [1] lean controls and individuals with obesity prior to bariatric surgery and [2] lean controls and individuals with obesity post-operatively; paired t-test was used for the [3] pre- and post-surgery comparison. When data were not normally distributed, Mann-Whitney U test was used for comparisons [1] and [2], and Wilcoxon matched-pairs signed rank test for comparison [3]. Fisher’s exact test was used to compare sex and comorbidity proportions between groups. Spearman’s rank correlation test was used to interrogate statistical significance in correlation matrices. Results were considered statistically significant at *p* < 0.05.

## Results

To address how the B cell compartment, inflammatory and metabolic parameters are affected by obesity and bariatric surgery, individuals with obesity undergoing bariatric surgery [25] were recruited pre-operatively and followed up 6 months after surgery and age- and sex- matched lean individuals with BMI < 25 were recruited as controls (referred to here as lean). See Methods for full participant inclusion and exclusion criteria. The demographic and clinical characteristics of the cohort are summarised in Table 1. No significant differences were observed in the presence of type 2 diabetes between the groups, while hypertension was present at a significantly higher frequency in individuals with obesity before surgery compared to lean controls and individuals post-bariatric surgery. As expected both weight and BMI of individuals with obesity prior to bariatric surgery was significantly higher compared to post-surgery and to lean controls; BMI and weight were also significantly higher when comparing individuals following bariatric surgery to lean controls.

**Table 1:** Demographic and clinical characteristics of study participants.

	Lean (*n* = 18)	Ob-pre (*n* = 21)	Ob-post (*n* = 17)	*p*-value		
				Lean vs Ob-Pre	Lean vs Ob-Post	Obese Pre- vs Ob-Post
Sex (F/M)	13/5	16/5	13/4	>0.9999	>0.9999	>0.9999
Age	43 (31.75–52)	49 (38–54.5)	50 (38–55)	0.1942	0.1040	n/a
BMI (kg/m^2^)	23.05 (21.65–24.38)	39.65 (36.60–44.38)	29.80 (27.85–33.70)	<0.0001	<0.0001	<0.0001
Weight (kg)	64.5 (60–73.25)	112.5 (96.0–131.5)	86.1 (71.4–100.8)	<0.0001	<0.0001	<0.0001
Excess weight (kg)	n/a	46.72 (29.69–51.74)	15.36 (7.36–23.05)	n/a	n/a	<0.0001
Weight loss (kg) | Excess weight loss (%)	n/a	n/a	26.4 (22–32) | 66.75 (52.23–86.72)	n/a	n/a	n/a
Type 2 diabetes	0	1	1	>0.9999	0.4857	>0.9999
Hypertension	1	11	2	0.0019	0.6026	0.0151

Age, BMI, weight data are median with IQR.

**Fig. 1:**
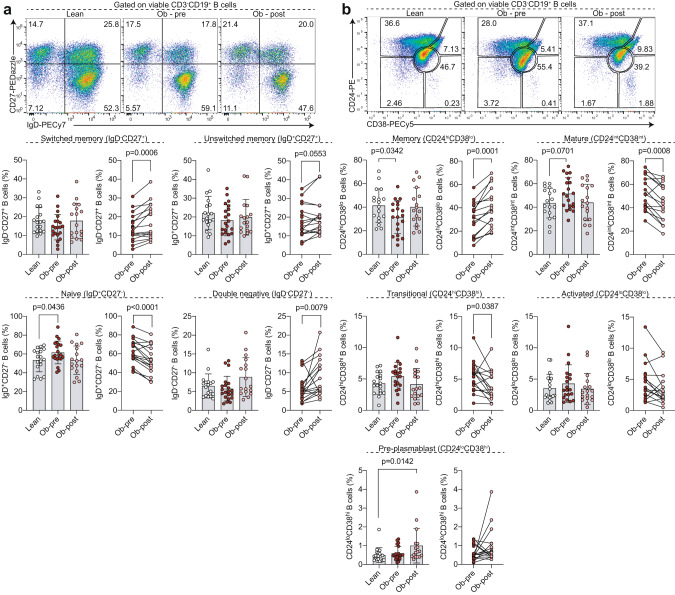
Distinct peripheral blood B cell profile following bariatric surgery compared to pre-operation and to lean controls. Representative flow cytometry plots and summary graphs demonstrating B cell distribution using the (**a**) the IgD- and CD27-based and (**b**) CD24- and CD38-based classification systems in lean controls and individuals with obesity prior to (Ob-pre) and 6 months following (Ob-post) bariatric surgery. Data are mean ± SD and each circle represents a study participant. For normally distributed IgD^-^CD27^+^, IgD^+^CD27^-^, CD24^hi^CD38^lo^, CD24^int^CD38^int^, CD24^hi^CD38^hi^ B cell populations independent samples t-test was used to compare [1] lean controls and individuals with obesity prior to bariatric surgery and [2] lean controls and individuals with obesity post-operatively; paired t-test was used for the [3] pre- and post-surgery comparison. For non-normally distributed IgD^+^CD27^+^, IgD^-^CD27^-^, CD24^lo^CD38^lo^, CD24^lo^CD38^hi^ B cell subsets Mann-Whitney U test was used for comparisons of lean and pre-surgery and lean and post-surgery, and Wilcoxon matched-pairs signed rank test for comparison between pre- and post-surgery samples.

We then carried out detailed profiling of PBMCs using two different well-established gating/classification strategies that allow to distinguish B cell populations based on IgD and CD27 and CD24 and CD38 expression [16, 27]. This strategy enabled us to identify the following populations within the CD19^+^ B cell compartment: IgD^+^CD27^-^ naïve, IgD^-^CD27^+^ class-switched and IgD^+^CD27^+^ unswitched memory, IgD^-^CD27^-^ DN cells, CD24^hi^CD38^hi^ transitional, CD24^int^CD38^int^ mature, CD24^hi^CD38^lo^ total memory, CD24^lo^CD38^lo^ activated B cells, and CD24^int^CD38^hi^ pre-plasmablasts. Of note, the populations identified by these gating strategies are overlapping, but were chosen to enable comparisons with previously published data [12, 22]. As reported previously [12, 22], we found a significant expansion of IgD^+^CD27^-^ naïve B cells in individuals with obesity compared to lean controls, which was normalised to control levels following surgery (Fig. 1a). Assessment of DN B cell frequency demonstrated that in our cohort there was a comparable frequency of DN B cells between healthy controls and individuals with obesity as well as between individuals with obesity pre- and post-operatively. We observed that CD24^hi^CD38^lo^ total memory B cells were reduced in individuals living with obesity compared to lean controls, but their frequency was restored post-operatively (Fig. 1b). The obesity-associated reduction in the memory compartment appeared to not be specifically associated with class-switching, as there was no difference between groups when comparing IgD^-^CD27^+^ class-switched and IgD^+^CD27^+^ unswitched memory B cell subsets (Fig. 1a).

**Fig. 2:**
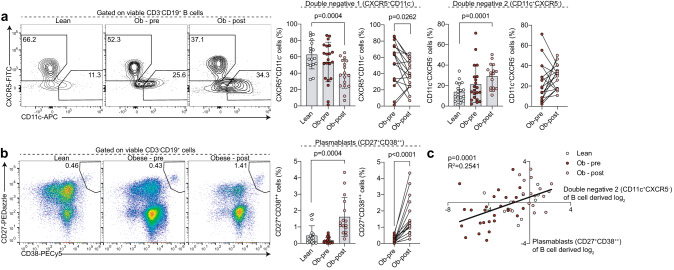
Expansion of CD11c^+^CXCR5^-^IgD^-^CD27^-^ DN2 B cells and CD27^+^CD38^++^ plasmablasts in the peripheral blood following bariatric surgery. Representative flow cytometry plots and summary graphs demonstrating (**a**) the distribution of IgD^-^CD27^-^ B cells into CXCR5^+^CD11c^-^ DN1 and CD11c^+^CXCR5^-^ DN2 subsets, (**b**) CD27^+^CD38^++^ plasmablast frequency and (**c**) linear regression analysis of log2-transformed DN2 versus CD27^+^CD38^++^ plasmablast frequencies of total B cell-derived cells in lean controls and individuals with obesity prior to (Ob-pre) and 6 months following (Ob-post) bariatric surgery. Data are mean ± SD and each circle represents a study participant. (**a**, **b**) For normally distributed CXCR5^+^CD11c^-^ DN1 independent samples t-test was used to compare [1] lean controls and individuals with obesity prior to bariatric surgery and [2] lean controls and individuals with obesity post-operatively; paired t-test was used for the [3] pre- and post-surgery comparison. For non-normally distributed CD11c^+^CXCR5^-^ DN2 and CD27^+^CD38^++^ plasmablasts Mann-Whitney U test was used for comparisons of lean and pre-surgery and lean and post-surgery, and Wilcoxon matched-pairs signed rank test for comparison between pre- and post-surgery samples, (**c**) Spearman’s rank correlation test.

To further interrogate how functional differentiation pathways are affected by obesity and bariatric surgery, we next assessed CXCR5^+^CD11c^-^IgD^-^CD27^-^ DN1 and CD11c^+^CXCR5^-^IgD^-^CD27^-^ DN2 B cell subset as well as CD27^+^CD38^++^ plasmablast frequencies. Following surgery patients had elevated DN2 frequency compared to lean controls and a reciprocal reduction in DN1 B cells (Fig. 2a). There was a trend for lower DN1 and increased DN2 B cell frequency in pre-surgery samples from individuals with obesity when comparing to lean controls. Similarly to DN2 B cells, we also found that CD27^+^CD38^++^ plasmablasts were expanded post-operatively compared to both lean controls and pre-surgery samples (Fig. 2b). Interestingly, as previously reported in the context of SARS-CoV-2 infection [21], we found a positive correlation between DN2 B cells and plasmablasts in this study population, suggesting a lineage relationship between these two subsets (Fig. 2c). Further analysis of the B cell compartment in a subset of study participants showed that DN2 B cells expressed high levels of T-bet, and low levels of CD21 and FcRL4 (Suppl. Figure 1), suggesting substantial phenotypic overlap with DN2 B cells characterised by others [17, 20, 28]. We did not observe differences in DN2 B cell phenotype between lean controls, and people with obesity pre- and post-bariatric surgery; this is consistent with observations in previous studies reporting that DN2 B cells are present in healthy controls at lower frequencies, but are expanded in SLE [17] and severe SARS-CoV-2 infection [20]. Of note, post-surgery expansion of DN2 B cells and plasmablasts was apparently not linked with global changes in antibody classes; total immunoglobulin (Ig) M, G, A levels were comparable between lean controls, and individuals pre- and post-bariatric surgery (Suppl. Fig. 2)

**Fig. 3:**
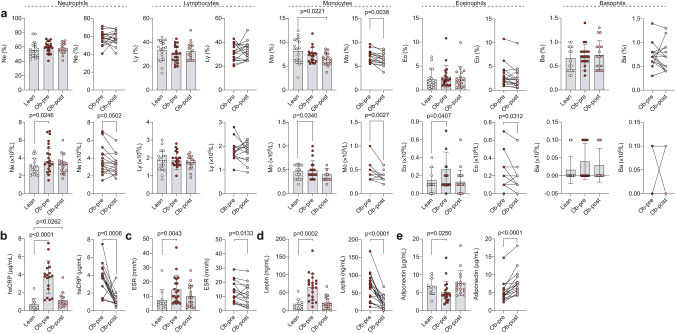
Obesity-associated inflammatory profile in the peripheral blood is attenuated following bariatric surgery. Graphs showing (**a**) leukocyte population frequency (top) and absolute count (bottom), (**b**) hsCRP, (**c**) ESR, (**d**) leptin and (**e**) adiponectin in lean controls and individuals with obesity prior to (Ob-pre) and 6 months following (Ob-post) bariatric surgery. Data are mean ± SD and each circle represents a study participant. For normally distributed neutrophil and lymphocyte frequencies and absolute counts, and leptin independent samples t-test was used to compare [1] lean controls and individuals with obesity prior to bariatric surgery and [2] lean controls and individuals with obesity post-operatively; paired t-test was used for the [3] pre- and post-surgery comparison. For non-normally distributed monocyte, eosinophil and basophil frequencies and absolute counts, hsCRP, ESR and adiponectin Mann-Whitney U test was used for comparisons of lean and pre-surgery and lean and post-surgery, and Wilcoxon matched-pairs signed rank test for comparison between pre- and post-surgery samples.

Considering the difference in the proportion of inflammation-associated B cell subsets post-bariatric surgery, we next wanted to assess the overall inflammatory profile in our cohort. We first interrogated major leukocyte subset frequencies and absolute numbers. Monocyte frequency, while comparable between individuals with obesity pre-bariatric surgery and lean controls, was reduced post-bariatric surgery compared to both lean controls and pre-operative samples (Fig. 3a, top row). We did not observe other alterations to leukocyte subset frequencies between the groups. More wide-spread obesity-associated changes were present in leukocyte absolute numbers (Fig. 3a, bottom row). Specifically, there was an obesity-associated expansion in neutrophil, monocyte and eosinophil absolute numbers pre-bariatric surgery which was restored to lean control levels post-operatively (Fig. 3a, bottom row). Individuals living with obesity also presented with elevated markers of inflammation in peripheral blood compared to lean controls, with increased acute phase reactant C-reactive protein (CRP) and erythrocyte sedimentation rate (ESR) (Fig. 3b, c). While both CRP levels and ESR were significantly reduced following bariatric surgery, post-operatively CRP levels remained significantly elevated compared to lean controls. Adipokines, such as leptin and adiponectin, are an essential link between metabolism and immune function, but are dysregulated in obesity, contributing to low-grade inflammation [29]. In agreement with published data, obesity was associated with elevated leptin (Fig. 3d) and decreased adiponectin (Fig. 3e) compared to lean controls [30, 31]. Similarly to other inflammatory parameters, the levels of adipokines were normalised to lean levels at 6 months following bariatric surgery.

**Fig. 4:**
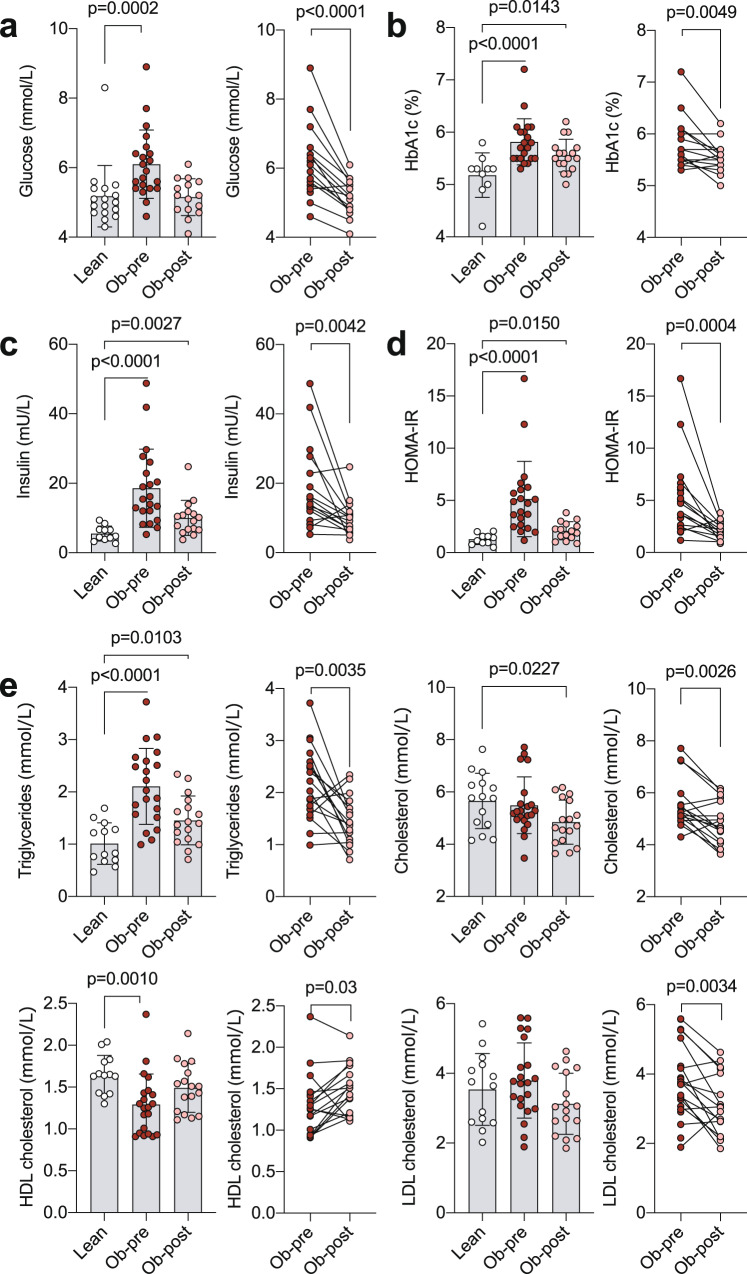
Normalisation of metabolic profile in individuals with obesity post-bariatric surgery. Graphs showing (**a**) glucose, (**b**) HbA1c, (**c**) insulin, (**d**) HOMA-IR and (**e**) lipids in lean controls and individuals with obesity prior to (Ob-pre) and 6 months following (Ob-post) bariatric surgery. Data are mean ± SD and each circle represents a study participant. For normally distributed triglycerides and LDL cholesterol independent samples t-test was used to compare [1] lean controls and individuals with obesity prior to bariatric surgery and [2] lean controls and individuals with obesity post-operatively; paired t-test was used for the [3] pre- and post-surgery comparison. For non-normally distributed glucose, HbA1c, insulin, HOMA-IR, total and HDL cholesterol Mann-Whitney U test was used for comparisons of lean and pre-surgery and lean and post-surgery, and Wilcoxon matched-pairs signed rank test for comparison between pre- and post-surgery samples.

Due to the intimate connection between inflammation and metabolic parameters and the extensively-reported benefits of bariatric surgery to the metabolic health of patients [7–10], we next interrogated the metabolic profile of lean individuals and people with obesity pre- and post-bariatric surgery. These data demonstrated that the elevated levels of glucose observed in individuals with obesity pre-surgery, were comparable to lean controls post-operatively (Fig. 4a). These data were supported by the assessment of HbA1c, a measure glucose metabolism over the preceding 2-3 months, insulin levels and HOMA-IR scores, all three of which were elevated in individuals living with obesity pre-surgery and significantly reduced post-operatively (Fig. 4b–d). Nevertheless, HbA1c, insulin, and HOMA-IR post-operatively were still significantly higher compared to lean controls. As well as glucose metabolites, measurement of blood lipids can be used as an assessment of metabolic health. Assessment of blood triglycerides, cholesterol, high density lipoprotein (HDL) and low-density lipoprotein (LDL) demonstrated that the heightened levels of these lipids observed in individuals with obesity pre-operatively were also mostly normalised to lean levels following surgery (Fig. 4e).

**Fig. 5:**
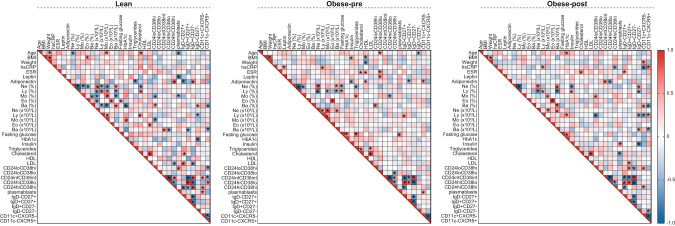
Inflammatory, metabolic and B cell correlations found in lean individuals are mostly lost in obesity and not restored post-operatively. Heatmaps demonstrating correlations between indicated immunometabolic parameters and B cells in lean controls and individuals with obesity prior to (Ob-pre) and 6 months following (Ob-post) bariatric surgery. * indicates all *p* < 0.05 (Spearman’s rank correlation test).

Finally, to begin to build a more comprehensive understanding of the relationships between the B cell compartment, inflammatory and metabolic parameters we carried out correlation analysis between these parameters in lean individuals and people with obesity pre- and post-bariatric surgery (Fig. 5). Some parameters, as expected, displayed significant correlations in all three groups, such as neutrophil frequency and absolute count of neutrophils, BMI and weight, and cholesterol and LDL (Fig. 5). Furthermore, B cell subsets defined by CD24 and CD38 expression correlated with their matched populations defined by IgD and CD27 expression in all three groups. For example, CD24^int^CD38^int^ mature naïve B cells correlated with IgD^+^CD27^-^ naïve B cells and CD24^hi^CD38^lo^ memory B cells correlated with IgD^+^CD27^+^ unswitched memory B cells in all three groups. When analysing differences between groups, we observed that correlations between lymphocyte, monocyte and basophil frequencies and their respective absolute numbers that were present in lean individuals, were lost in obesity and restored following surgery. When interrogating correlations between metabolic parameters and B cell subsets in lean individuals we observed that unlike the negative correlation between leptin and CD27^+^CD38^++^ plasmablasts in lean individuals, in post-surgery patients we observed a trend for a positive correlation. We further identified a significant positive correlation between adiponectin and both CD24^hi^CD38^hi^ transitional B cells and IgD^+^CD27^-^ naïve B cells in lean individuals. These correlations were absent in individuals with obesity and not restored following surgery. However, following bariatric surgery, adiponectin did correlate with CD24^int^CD38^int^ mature naïve B cells similarly to lean controls. Thus it is possible that an interaction between adiponectin and naïve B cells is disrupted in obesity when adiponectin levels are low, but is restored to lean control levels with the elevation of adiponectin following bariatric surgery. Together, these underscore the profound differences in B cell subsets between lean people and individuals with obesity prior to and following bariatric surgery suggesting that lean, obesity pre- and post-surgery are unique immunometabolic states.

## Discussion

Here we have carried out an in-depth assessment of peripheral blood B cell profile, obesity-associated inflammation and metabolic parameters in lean individuals, and people with obesity pre- and post-bariatric surgery. These data demonstrate that whilst there is a general normalisation in metabolic and inflammatory parameters in individuals with obesity post-surgery, distinct B cell profiles are associated with healthy weight, obesity, and the post-bariatric surgery state. Our data further demonstrate that correlations observed between metabolic and inflammatory parameters and B cell subsets in lean individuals are lost during obesity and not restored by 6 months post-bariatric surgery. These findings add to the evidence base that once the intricate interplay between metabolic/inflammatory parameters and immune cell function is disrupted even profound changes to metabolic health and reduction in inflammation, such as following bariatric surgery, may not result in the full recovery of adaptive immune function associated with healthy weight.

More specifically, in this study the interrogation of peripheral B cell subsets using surface phenotypes allowed us to gain insight into how the functionality of the B cell compartment is affected in people with obesity pre- and post-bariatric surgery. One of our most prominent observations was that the disrupted naïve and memory cell balance – with expansion of IgD^+^CD27^-^ naïve B cells and reduction in CD24^hi^CD38^lo^ memory B cells – in obesity was normalised following bariatric surgery. Notably, obesity has been previously shown to be associated with a reduction in memory B cells [22] and naïve B cell expansion [12, 22], and our data further add to these findings by demonstrating that bariatric surgery is able to rescue this ‘imbalance’ in individuals with obesity. Multiple pathways may lead to obesity-associated dysregulation of naïve and memory B cell balance. This includes alterations in B cell differentiation pathways by pro-inflammatory mediators and, potentially, migration of memory B cells to secondary inflammatory sites, specifically the adipose tissue. Previously Frasca et al. had reported significant expansion of IgD^-^CD27^-^ DN B cells in obesity [22]. However, like Wijngaarden et al., we did not observe a significant obesity-associated expansion of IgD^-^CD27^-^ DN B cells [12]. The reasons underlying the discrepancies between studies remain unclear and are likely due to differences in the core demographics such as age, sex and ethnicity. In our study, we were able to recruit a well-matched, and indeed for the pre- and post-bariatric surgery patients paired, patient cohort that was extremely homogenous ~90% female, <60 years old and not ethnically diverse, as it was recruited from a single centre in Latvia; a country with a population that is predominantly white eastern European. The strength of using this cohort is that the impact of bariatric surgery on immune and metabolic parameters could be assessed with relatively few clinical confounders. Importantly, the obesity-associated changes in B cell subsets described in this study are likely to contribute, at least in part, to infection and vaccination outcomes in individuals with obesity. For example, due to their superior proliferative and functional properties, memory B cells are important to guard against repeat exposure to pathogens as well as accelerated ‘evolution’ of the antibody response to novel variants [32]. Thus, reduction in the proportion of memory B cells is likely to directly affect pathogen clearance and vaccination-provided protection in obesity upon re-exposure to pathogens. Ostensibly the normalisation of memory and naive subsets following bariatric surgery would result in at least partial restoration of immune responsiveness; this will be addressed by studies exploring de novo immunity to pathogens and immunisation following surgery.

Although we did not observe any difference in the frequency of the IgD^-^CD27^-^ DN B cells between lean individuals and pre-bariatric surgery patients with obesity, we did find a marked increase in the frequency of inflammatory CD11c^+^CXCR5^-^IgD^-^CD27^-^ DN2 B cells and CD27^+^CD38^++^ plasmablasts post-bariatric surgery. In a subset of individuals, we also found that DN2 expressed T-bet, but lacked CD21 or FcRL4, a DN2 phenotype consistent with that previously identified by others [17, 28]. DN2 B cell phenotype was comparable in lean individuals and people with obesity pre- and post-surgery, which is in line with altered frequencies, but similar transcriptional profiles of DN2 B cells in patients with SLE compared to healthy controls [17]. Furthermore, the correlation between DN2 B cells and plasmablasts suggests that there is heighted EF B cell activation following surgery as previously shown in the context of COVID-19 [21]. The mechanism underlying the increase in B cells associated with the EF pathway is unclear. One possibility is that self-antigens released through adipocyte death during weight-loss could drive de novo extrafollicular responses through B cell receptor or toll-like receptor activation in the presence of cytokines (including, IFN-γ, IL-21) as postulated in autoimmunity, aging and severe SARS-CoV-2 infection [17, 18, 28, 33, 34]. Alternatively, considering that obese adipose tissue has been reported to be a reservoir of IgD^-^CD27^-^ DN B cells [35], it is tempting to speculate that the obese adipose provides a microenvironmental niche (with excess fatty acids, hypoxia, inflammasome activation) for the generation or recruitment of CD11c^+^CXCR5^-^IgD^-^CD27^-^ DN2 and that these are ‘liberated’ from adipose during weight loss following bariatric surgery. Indeed, studies have previously linked circulating classical monocytes with CD11c^+^ macrophages in adipose tissue [36], and based on these data, future experiments should address the provenance of CD11c^+^CXCR5^-^IgD^-^CD27^-^ DN2 cells and CD27^+^CD38^++^ plasmablasts in the context of bariatric surgery. Understanding the ontogeny, relative sensitivity to stimuli (e.g. antigens via BCR, TLR ligands, cytokines) and functional capacity of DN2 B cells that are expanded post-bariatric will be key for future studies.

With regards to the functional significance of the activation of the EF pathway following bariatric surgery, recent investigations of COVID-19 have suggested the EF response as the source of de novo autoimmunity in patients with severe disease, specifically anti-nuclear antigen (ANA) and anti-carbamylated protein antibodies were detected [21]. Indeed, a prospective study of 30 bariatric surgery patients found that 4 of them developed ANA at 10 months post-operatively [37]. Thus, heightened EF responses link the similar B cell phenotypes observed in COVID-19 patients, autoimmunity and post-bariatric surgery individuals.

Of note, an important limitation of this study is that at 6 months post-bariatric surgery the weight loss is likely to be on-going, peaking at approx. 12 months post-bariatric surgery [38]. This ongoing weight loss may then drive some peripheral B cell parameters despite normalisation of metainflammation. Accordingly, it would be informative to assess these cellular abnormalities at later time-points such as at least 24 months post-surgery upon the establishment of weight homeostasis. Furthermore, comparison to life-style interventions would enable to assess the impact of more subtle and gradual weight loss in the absence of surgery as has been previously carried out for cytokines and regulatory T cells [39].

This study identifies that the immune compartment 6 months following bariatric surgery is not the same as lean individuals, future research needs to establish the functional consequences of the phenotypic differences described here. What also remains unknown is whether changes following obesity are transient or permanent, and to date, no studies have compared outcomes in infectious disease or post-vaccination in post-bariatric individuals that retain a healthy weight. Our data demonstrate that many immune and metabolic parameters are normalised 6 months post-bariatric surgery, making surgery an attractive therapeutic avenue to normalise both immune and metabolic parameters associated with ‘meta-inflammation’. However, considering the high socioeconomic costs of obesity on world-wide heath systems and the growing number of individuals with obesity [40], understanding the long-term physiological changes associated with the post-obese state remains of critical importance; particularly further understanding of how new set-points acquired through bariatric surgery relate to understanding of immune activation and memory pathways established in lean individuals.

### Supplementary information

Supplemental material

## Data Availability

The data of this study are available from the corresponding author, KO, upon reasonable request.
