# Supplementary material for: Distinct B cell profiles characterise healthy weight and obesity pre- and post-bariatric surgery
Source: Int J Obes (Lond). 2023 Jul 18;47(10):970–8. doi: 10.1038/s41366-023-01344-y (PMC10511309; doi:10.1038/s41366-023-01344-y)
Supplement: Supplementary file 1 — Supplemental material [file 41366_2023_1344_MOESM1_ESM.pdf]

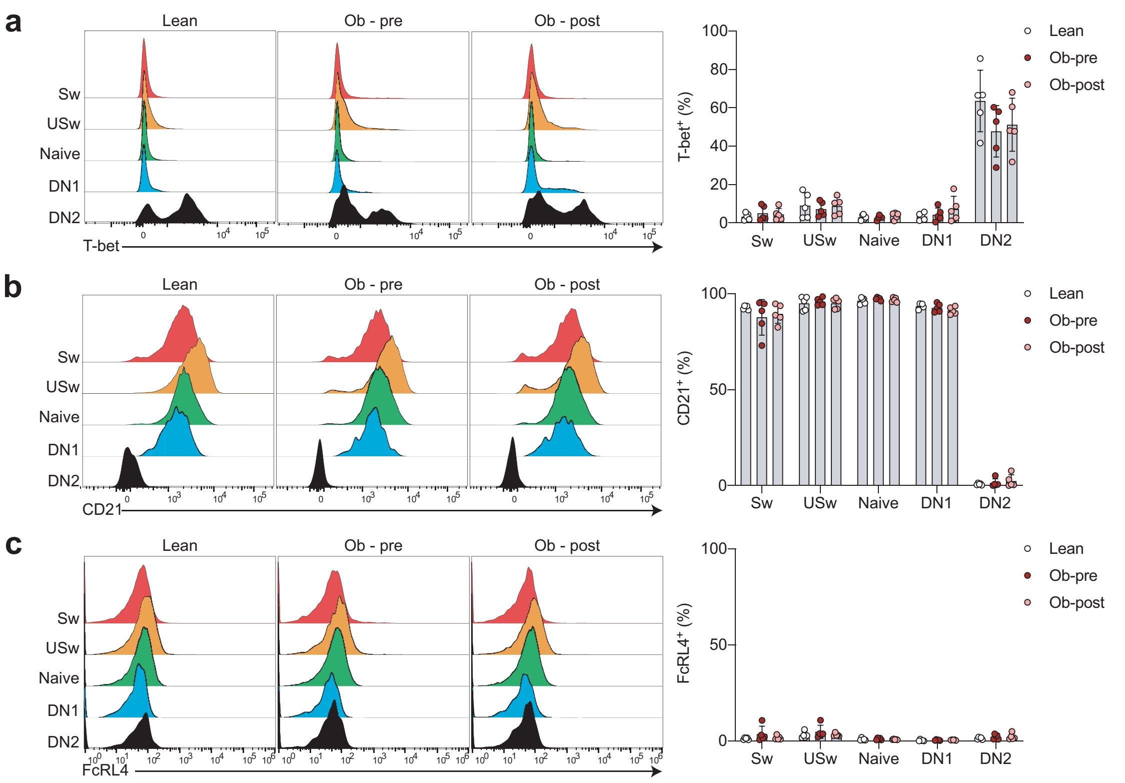

Supplementary Figure 1. Representative flow cytometry plots and summary graphs demonstrating **a** T-bet, **b** CD21, and **c** FcRL4 expression in switched (Sw) and unswitched (USw) memory, naïve, DN1 and DN2 B cell subsets in lean controls and individuals with obesity prior to (Ob-pre) and 6 months following (Ob-post) bariatric surgery. Data are mean±SD and each circle represents a study participant. **a-c** Two-way ANOVAs with Sidak multiple comparisons tests.

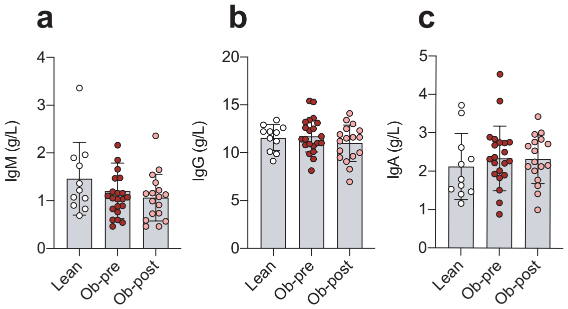

Supplementary Figure 2. Summary graphs of serum **a** IgM **b** IgG and **c** IgA in lean controls and individuals with obesity prior to (Ob-pre) and 6 months following (Ob-post) bariatric surgery. Data are mean±SD and each circle represents a study participant. For normally distributed IgM and IgG t test was used to compare (1) lean controls and individuals with obesity prior to bariatric surgery and (2) lean controls and individuals with obesity post-operatively; paired t test was used for the (3) pre- and post-surgery comparison. For non-normally distributed IgA Mann-Whitney U test was used for comparisons of lean and pre-surgery and lean and post-surgery, and Wilcoxon matched-pairs signed rank test for comparison between pre- and post-surgery samples.

Supplementary Table 1. Antibodies used in flow cytometry.

Antigen	Fluorochrome	Clone	Cat no	Supplier	Dilution
CD3	APC-Cy7	HIT3a	300318	BioLegend	1:20
CD11c	APC	3.9	301614	BioLegend	1:100
CD19	AF700	HIB19	557921	BD Biosciences	1:50

CD27	PE-Dazzle594	M-T271	356422	BioLegend	1:50
CD38	PE-Cy5	HIT2	303508	BioLegend	1:20
CXCR5	FITC	J252D4	356914	BioLegend	1:20
IgD	PE-Cy7	IA6-2	348210	BioLegend	1:20
CD24	PE	ML5	311106	BioLegend	1:50
T-bet	PE	eBio4B10 (4B10)	12-5825-82	Invitrogen	1:40
FcRL4	PE	413D12	340204	BioLegend	1:20
CD21	FITC	Bu32	354910	BioLegend	1:20
CD25	PE	M-A251	356104	BioLegend	1:20
